# Supplementary material for: A Comparative Assessment of Human and Chimpanzee iPSC-derived Cardiomyocytes with Primary Heart Tissues
Source: Sci Rep. 2018 Oct 17;8:15312. doi: 10.1038/s41598-018-33478-9 (PMC6193013; doi:10.1038/s41598-018-33478-9)
Supplement: Supplementary file 1 — Supplementary Figures [file 41598_2018_33478_MOESM1_ESM.pdf]

A Comparative Assessment of Human and Chimpanzee iPSC-derived Cardiomyocytes with Primary Heart Tissues

Bryan J. Pavlovic[1,*], Lauren E. Blake[1], Julien Roux[2,3], Claudia Chavarria[1], Yoav Gilad[1,4,*]

1. University of Chicago, Department of Human Genetics, Chicago, IL.
2. Department of Biomedicine, University of Basel, Basel, Switzerland.
3. Swiss Institute of Bioinformatics, 1015 Lausanne, Switzerland
4. University of Chicago, Department of Medicine, Chicago, IL.

[*]Correspondence should be addressed to Yoav Gilad (gilad@uchicago.edu) or Bryan J. Pavlovic (bjp@uchicago.edu).

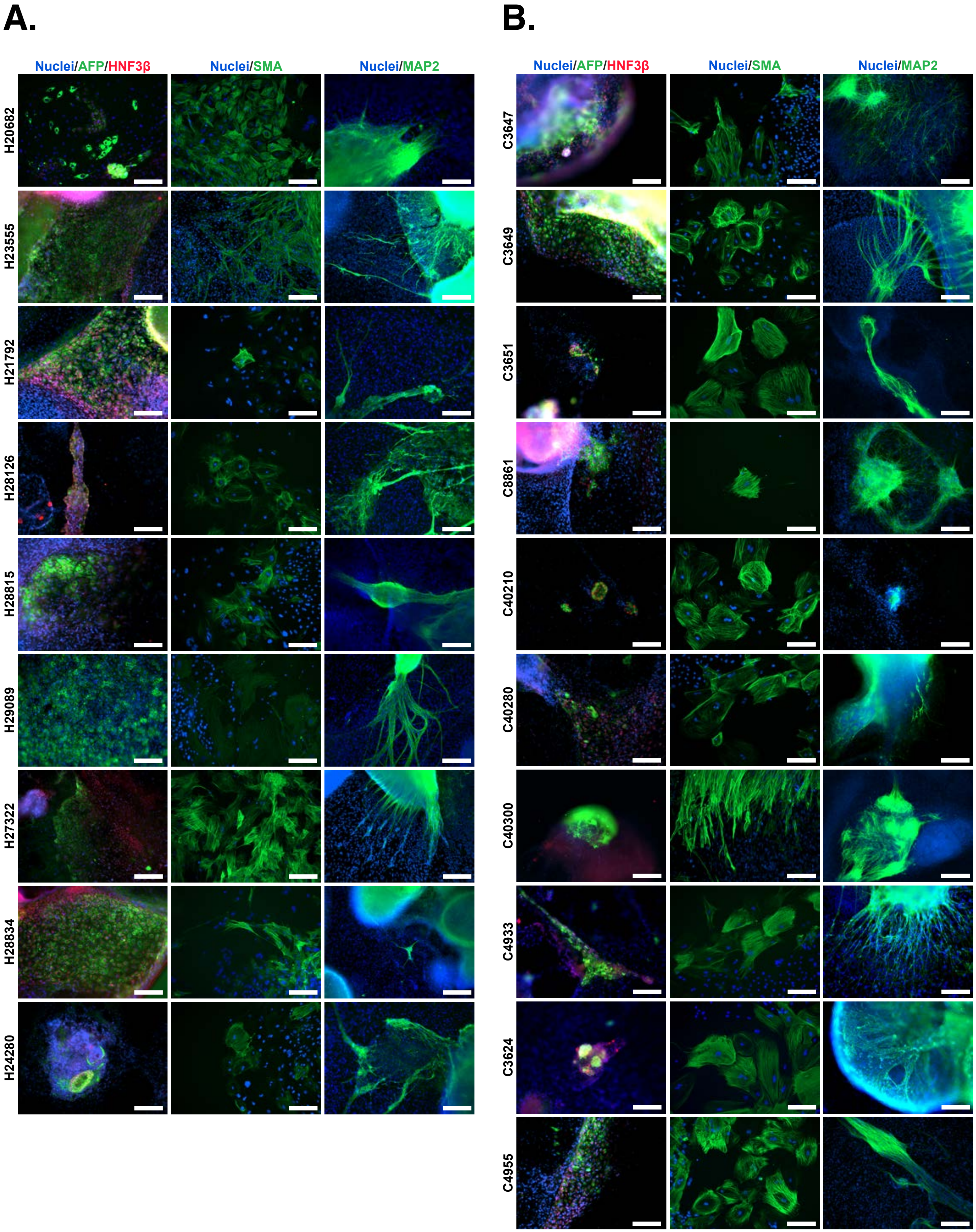

Supplemental Figure 1. Spontaneous differentiation assay of pluripotency for iPSC lines used in this study. A. Immunocytochemistry (ICC) staining of spontaneously differentiated embryoid bodies for Human iPSC lines. **B.** Immunocytochemistry (ICC) staining of spontaneously differentiated embryoid bodies for chimpanzee iPSC lines, antibodies identifying cell types derived from the three germ layers as indicated. Scale bar: 200 μM.

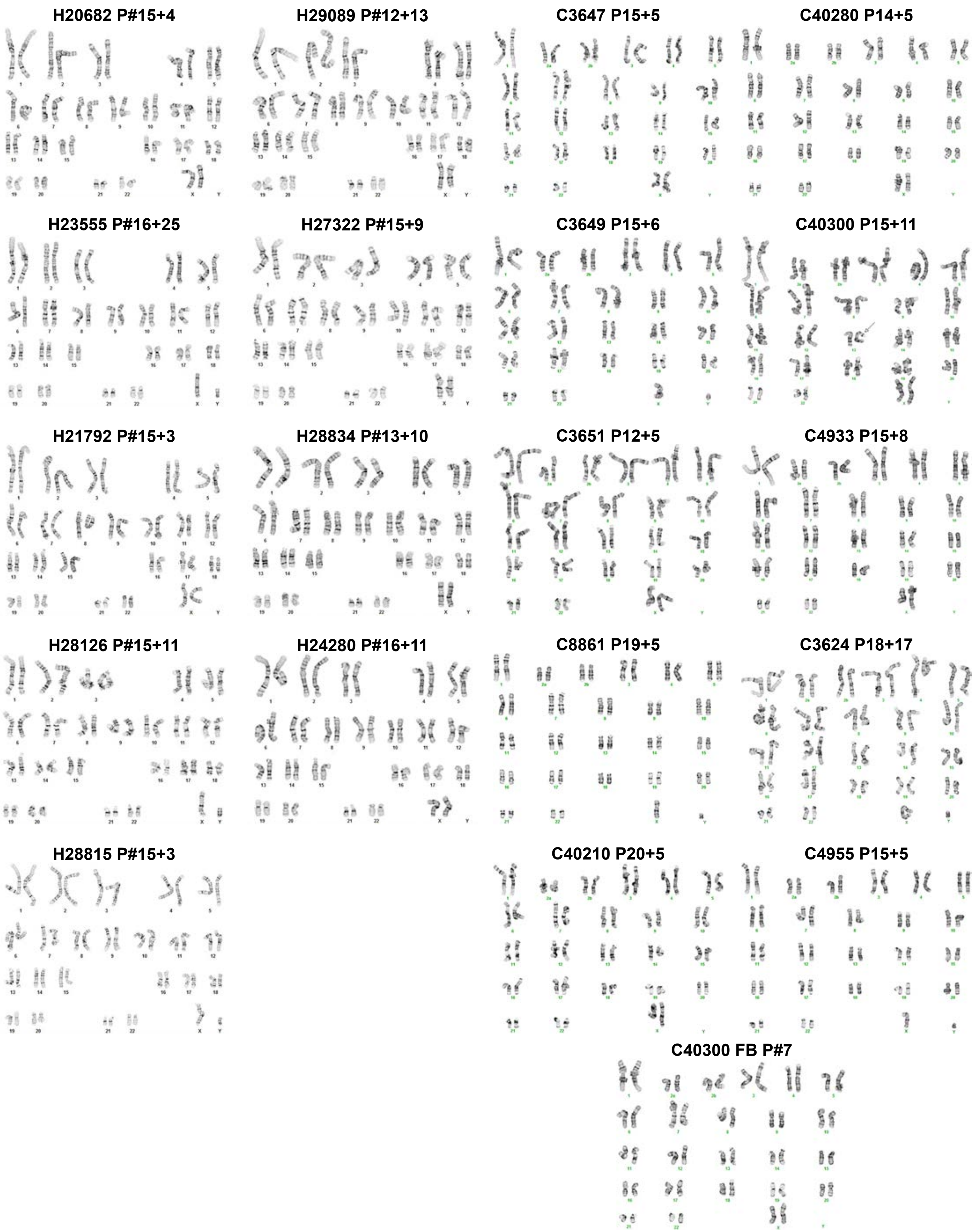

Supplemental Figure 2. Karyotypes for human and chimpanzee iPSC lines. Karyotypes for human (right) and chimpanzee lines (left) used in this study. We identified additional bands in the p-arms of one chromosome 13 homolog and one chromosome 18 homolog for chimpanzee iPSC line C3. Thus, we tested the source fibroblast line (C3 FB) to determine that these polymorphisms were normal polymorphisms and not formed de novo as a result of reprogramming.

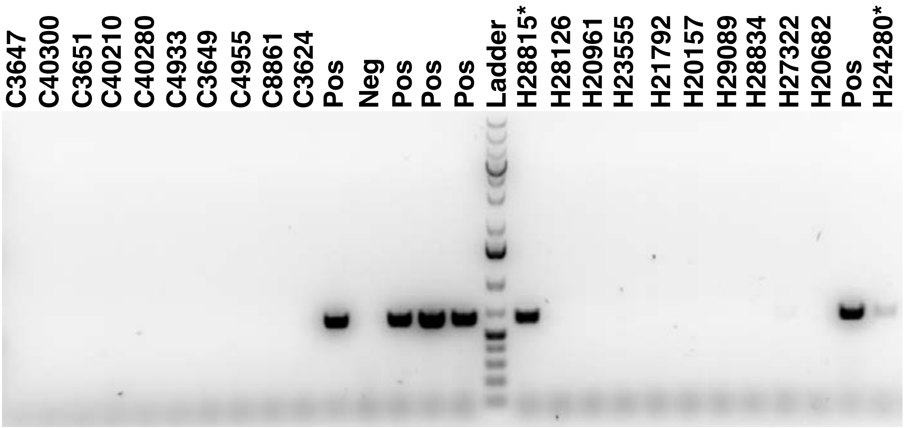

Supplemental Figure 3. PCR gel to test for exogenous episomal reprogramming vectors. PCR gel for all iPSC lines used for this study. Pos indicates reprograming vector positive DNA controls, Neg is a reprograming vector negative control. Human lines H28815 and H24280 demonstrate positive results for presence of reprogramming plasmid.

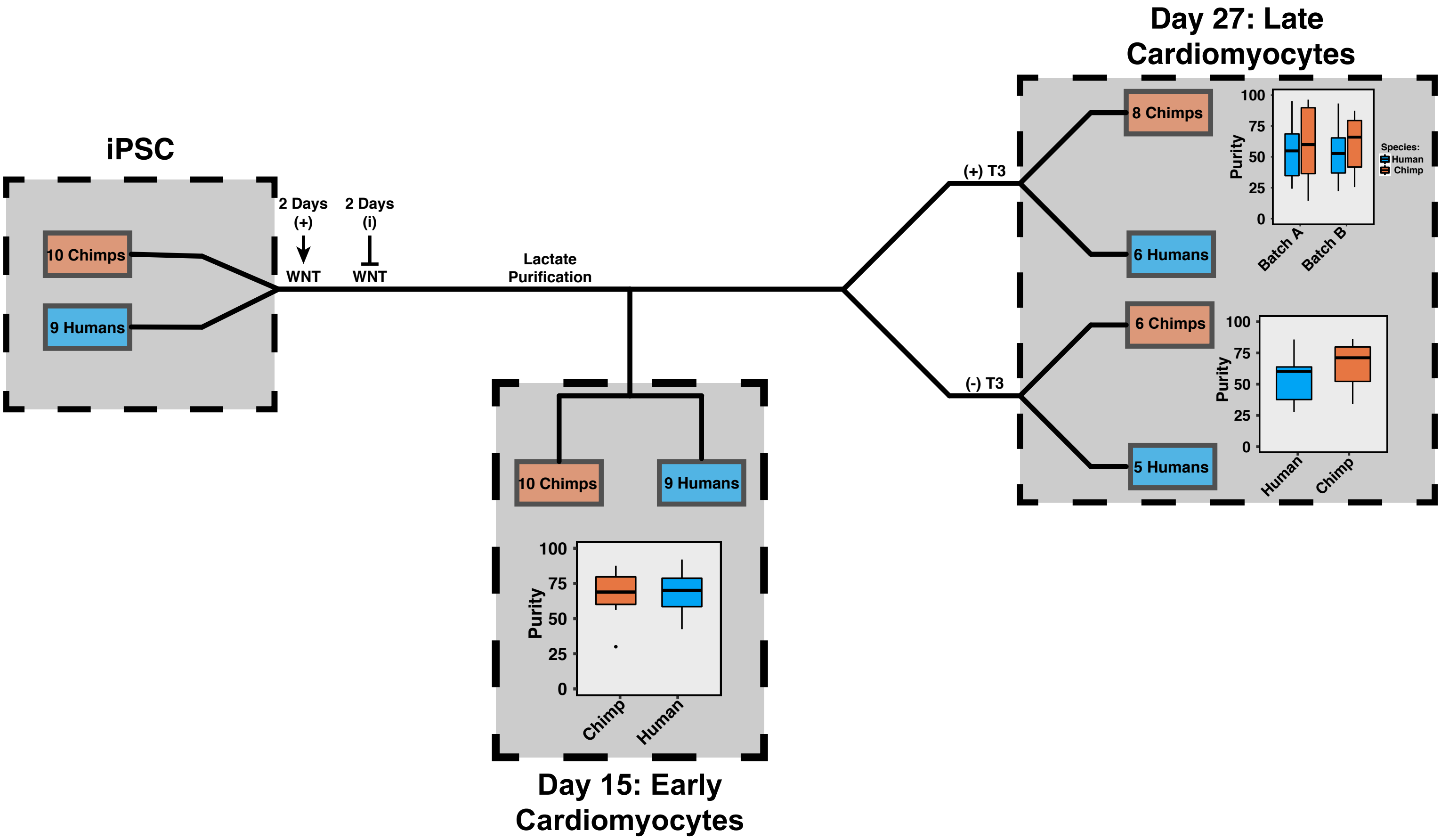

Supplemental Figure 4. Study design with iPSC-derived cardiomyocytes differentiation outline and summary of data collected. We induced differentiation from iPSC lines using a previously published protocol using WNT agonist for 2 days followed by WNT antagonism for 2 days. At day 15 we harvested RNA from early iPSC-derived cardiomyocytes after metabolic purification with lactic acid. Within a single batch, we collected 10 chimps and 9 human lines. Cells from a subset of individuals were replated and allowed to recover before starting treatment with T3 ((+)T3). A matched set of cells were cultured in parallel but not treated with T3 ((-)T3). We harvested RNA from (+)T3 and (-)T3 day 27 iPSC-derived cardiomyocytes within the same batch that was matched to the day 15 collection (batch B). We also collected another set of (+)T3 treated Day 27 iPSC-derived cardiomyocytes that did not have a matched (-)T3 or Day 15 set (denoted batch A). Purities obtained from dual positive (TNNI3+/TNNT2+) flow cytometry analysis for iPSC-derived cardiomyocytes samples are show in box plots for each set of samples and batches. Grey shaded boxes denote where sample collection occurred.

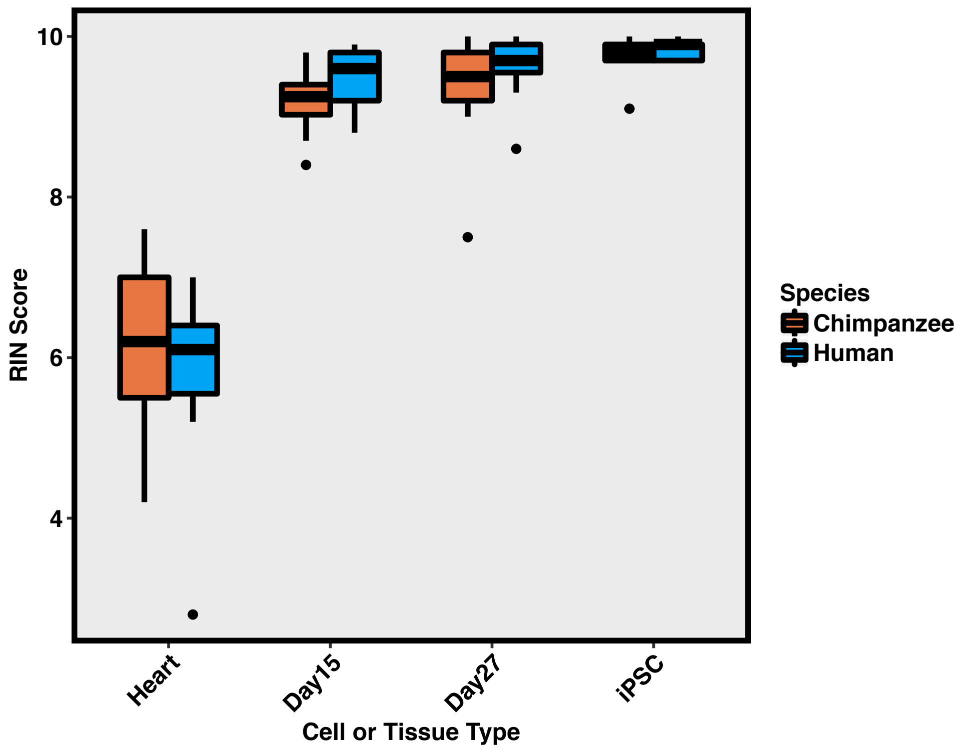

Supplemental Figure 5. RIN scores for all samples collected in this study. RIN scores separated by species and cell or tissue type for all samples collected in this study.

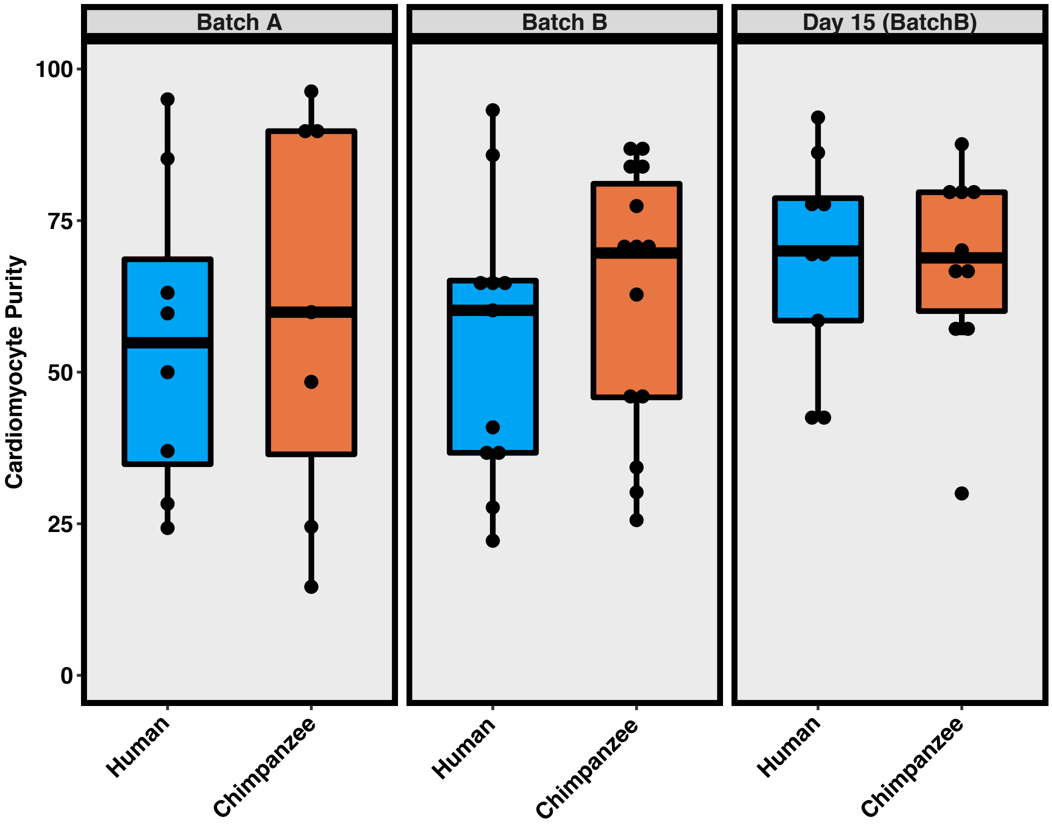

Supplemental Figure 6. Differentiation batch and purity are not associated with species. Distribution of purities for human and chimpanzee iPSC-derived cardiomyocytes for all samples collected in this study stratified by differentiation batch.

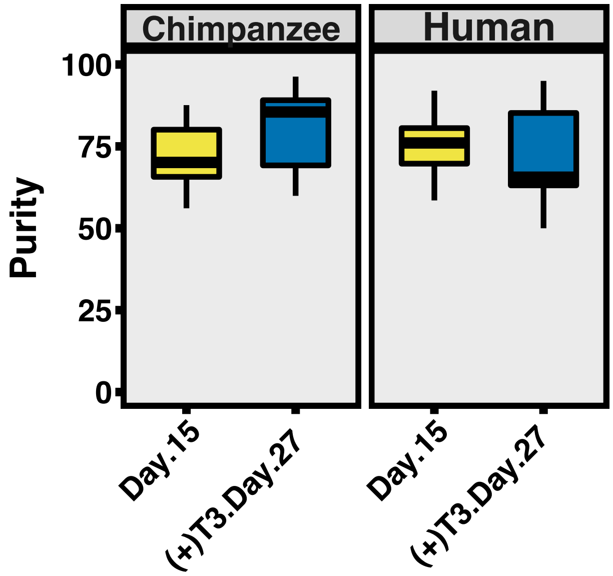

Supplemental Figure 7. Purity of samples used for analysis of genes that are under differential regulation within iPSCs, iPSC-derived cardiomyocytes and heart tissue. Box plots showing the purity of seven chimpanzee (left) and seven human (right) day 15 and day 27 iPSC-derived cardiomyocytes used for analysis, see Supplemental Table 1 for list of samples used.

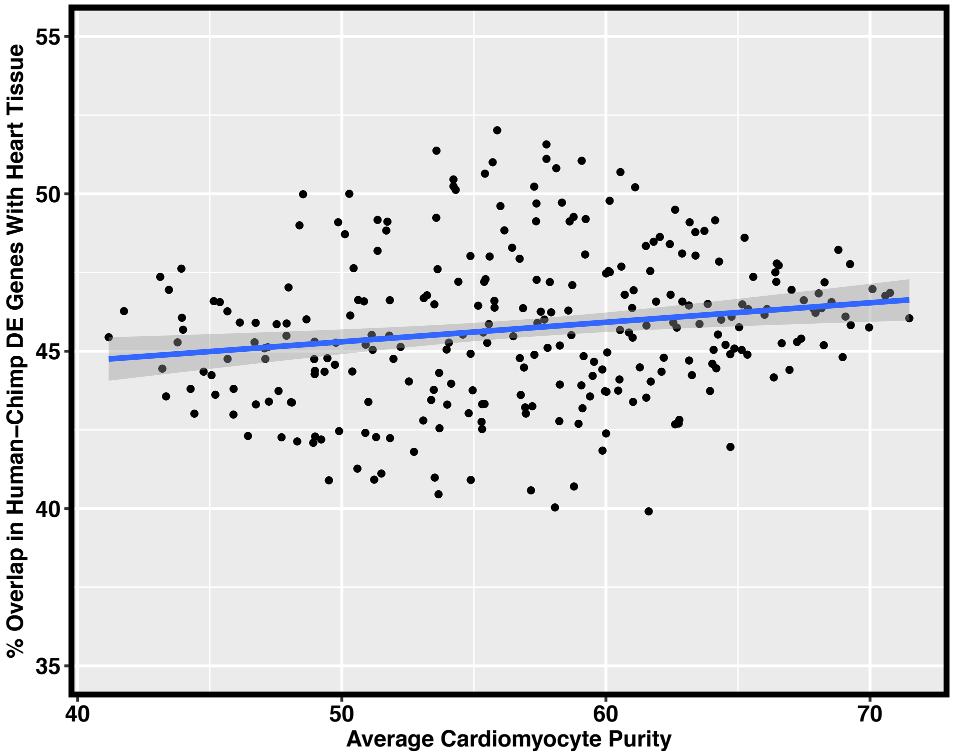

Supplemental Figure 8. Effect of purity on recapitulation of interspecies DE patterns using iPSC-derived cardiomyocytes. Scatter plot showing the average purity of sample set used on X axis and the percentage of human-chimpanzee DE genes identified on the Y axis. Regression line is shown for Y ~ X in blue with a 95% confidence interval in gray.

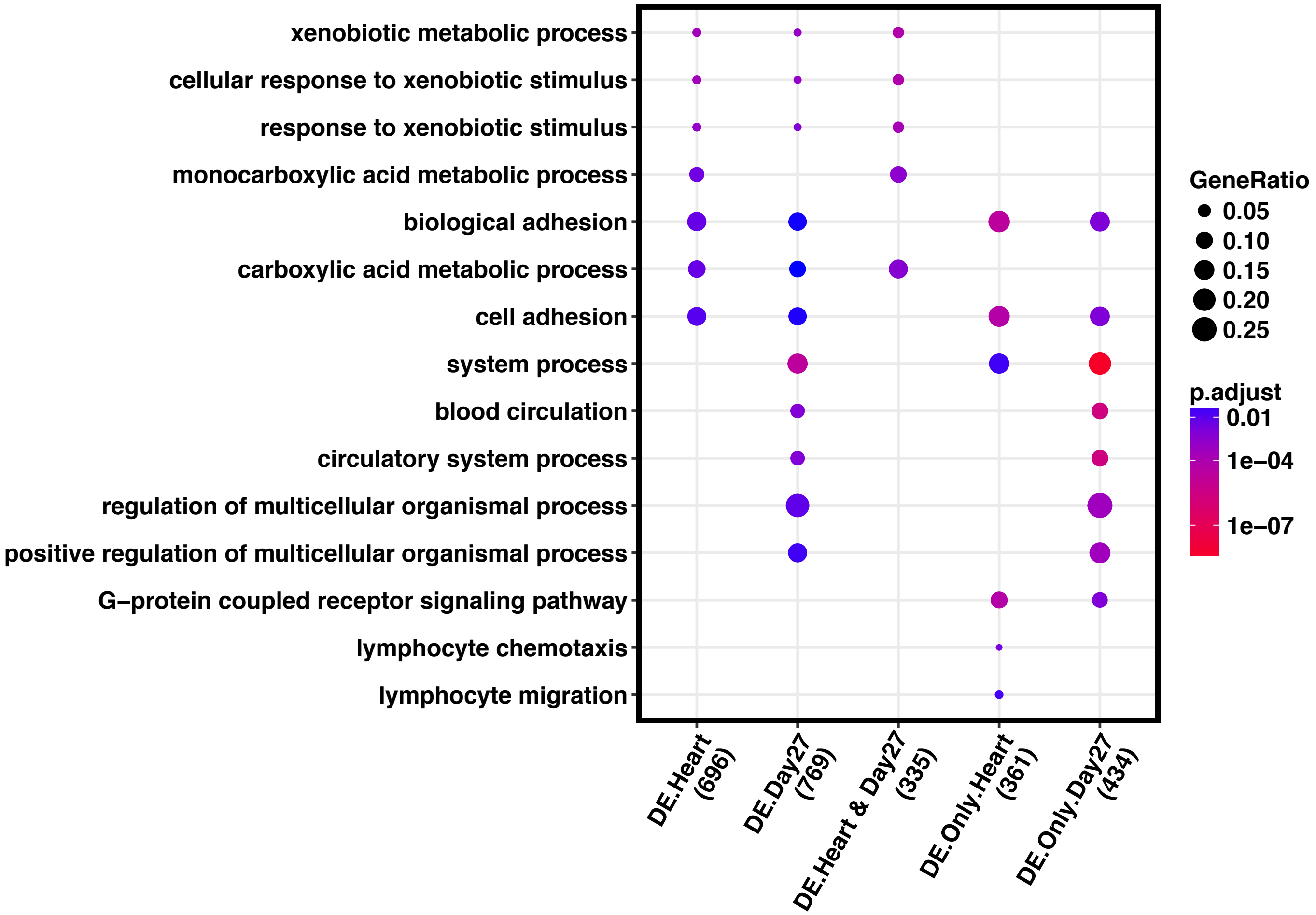

Supplemental Figure 9. Gene enrichment analysis for interspecies DE genes. The top enrichments in GO biological processes for each of the categories of genes is shown, gene list sizes are shown under the label for each gene set. Complete GO enrichment results are available in Supplemental Table 5.

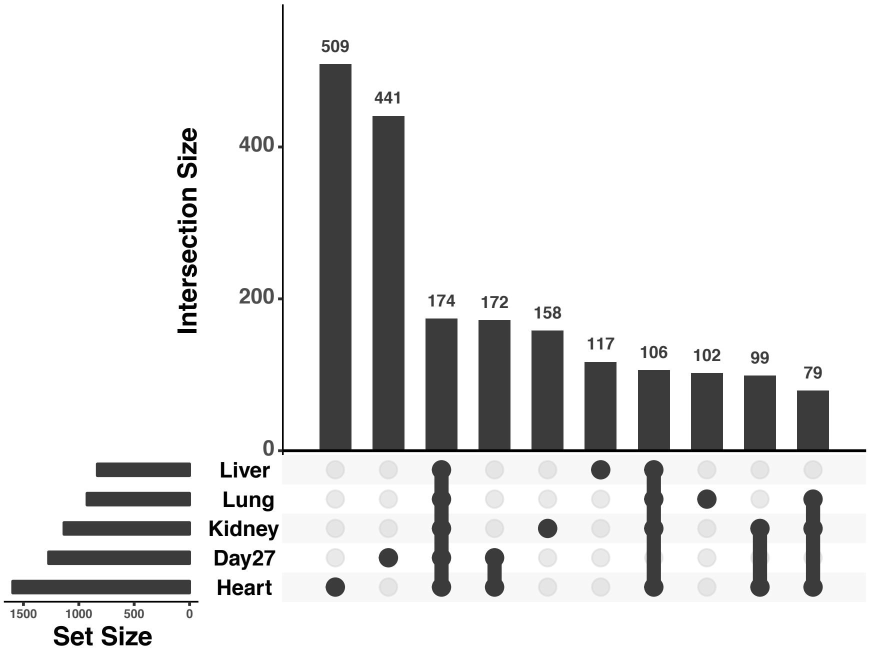

Supplemental Figure 10. Upsetr plot showing top 10 largest interspecies DE gene overlaps across multiple tissues and day 27 cardiomyocytes. Total set sizes are shown on the bottom left, overlaps are shown by links with a filled circle, and the bar plot above link shows size of a specific overlap. Plot generated using R package Upsetr (Lex et al. 2014).

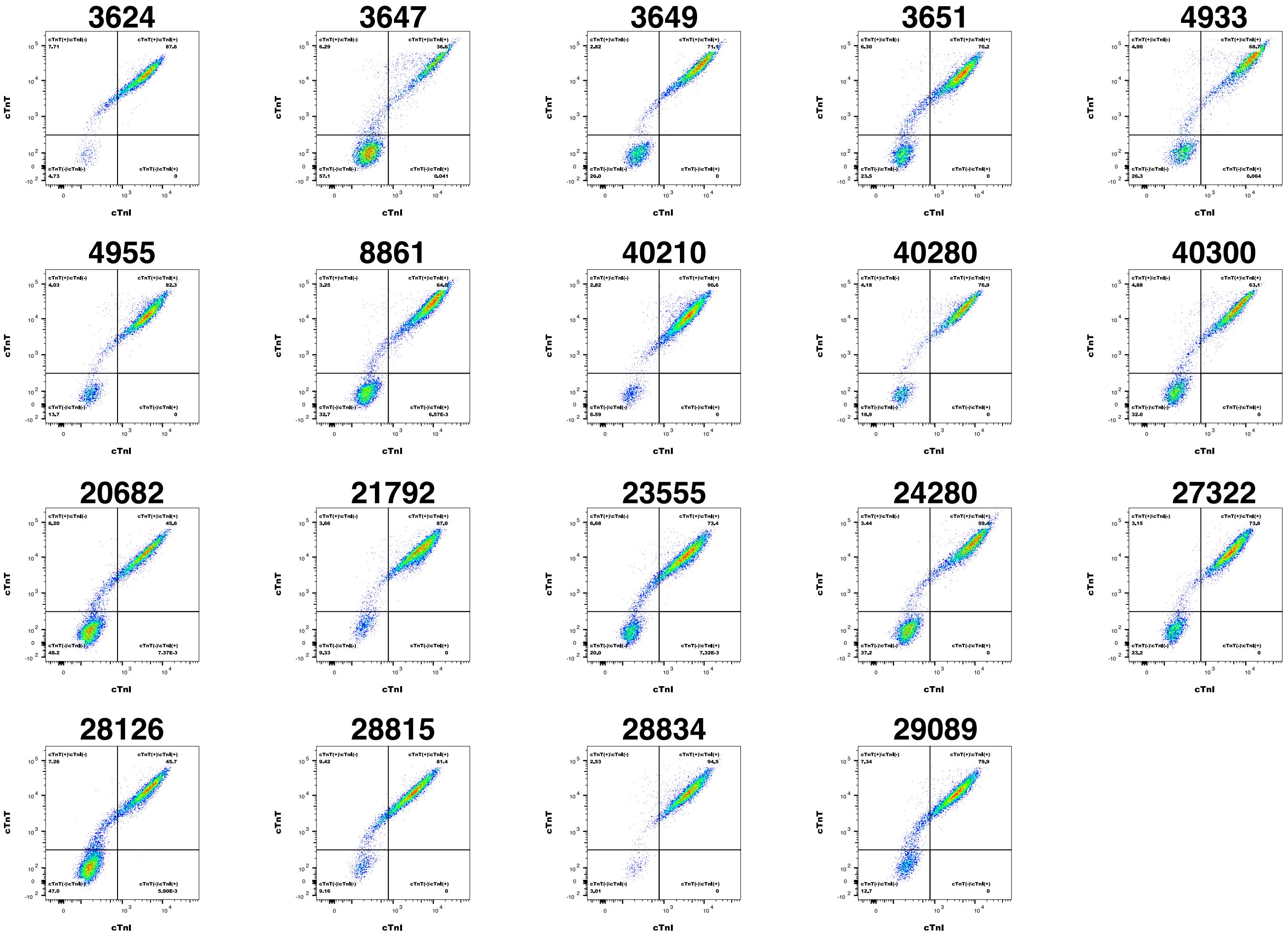

Supplemental Figure 11. Psuedocolor plots showing gating for iPSC-derived cardiomyocytes from batch B at Day 15. Gating and purities are shown for iPSC-derived cardiomyocyte samples collected in batch B at day 15. Plots and purities are shown with dead cells and debris already removed from analysis (methods). iPSC line identifiers designating different individuals are show above each plot, all line and sample info is included in Supplemental Table 1. Fluorescence intensity for cTnT (TNNT2-PE) is shown on the y-axis, fluorescence intensity for cTnI (TNNI3-A647) is shown on the x axis.

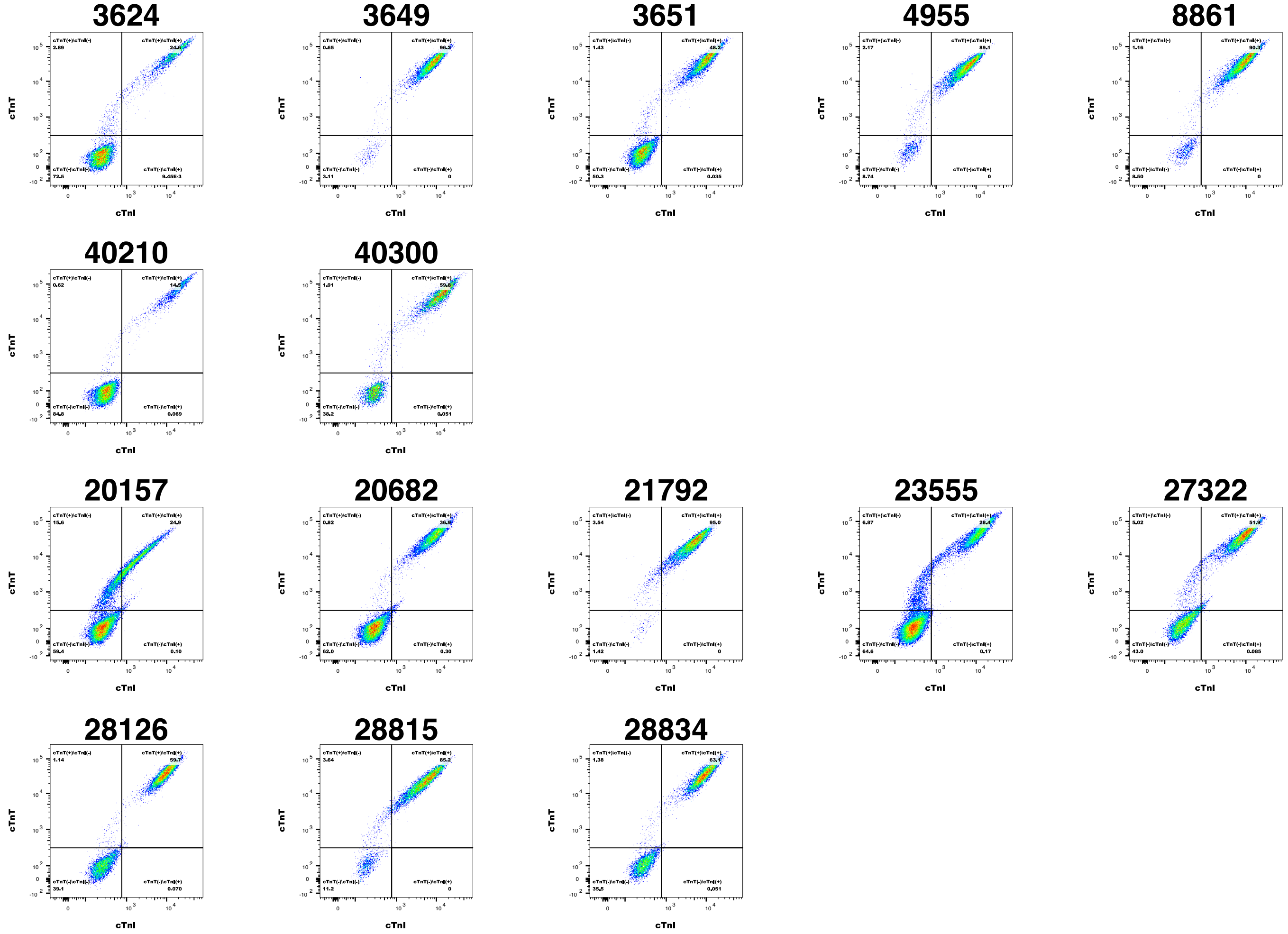

Supplemental Figure 12. Psuedocolor plots showing gating for iPSC-derived cardiomyocytes from batch A at Day 27. Gating and purities are shown for iPSC-derived cardiomyocyte samples collected in batch A at day 27 with T3 treatment. Plots and purities are shown with dead cells and debris already removed from analysis (methods). iPSC line identifiers designating different individuals are show above each plot, all line and sample info is included in Supplemental Table 1. Fluorescence intensity for cTnT (TNNT2-PE) is shown on the y-axis, fluorescence intensity for cTnI (TNNI3-A647) is shown on the x axis.

A.

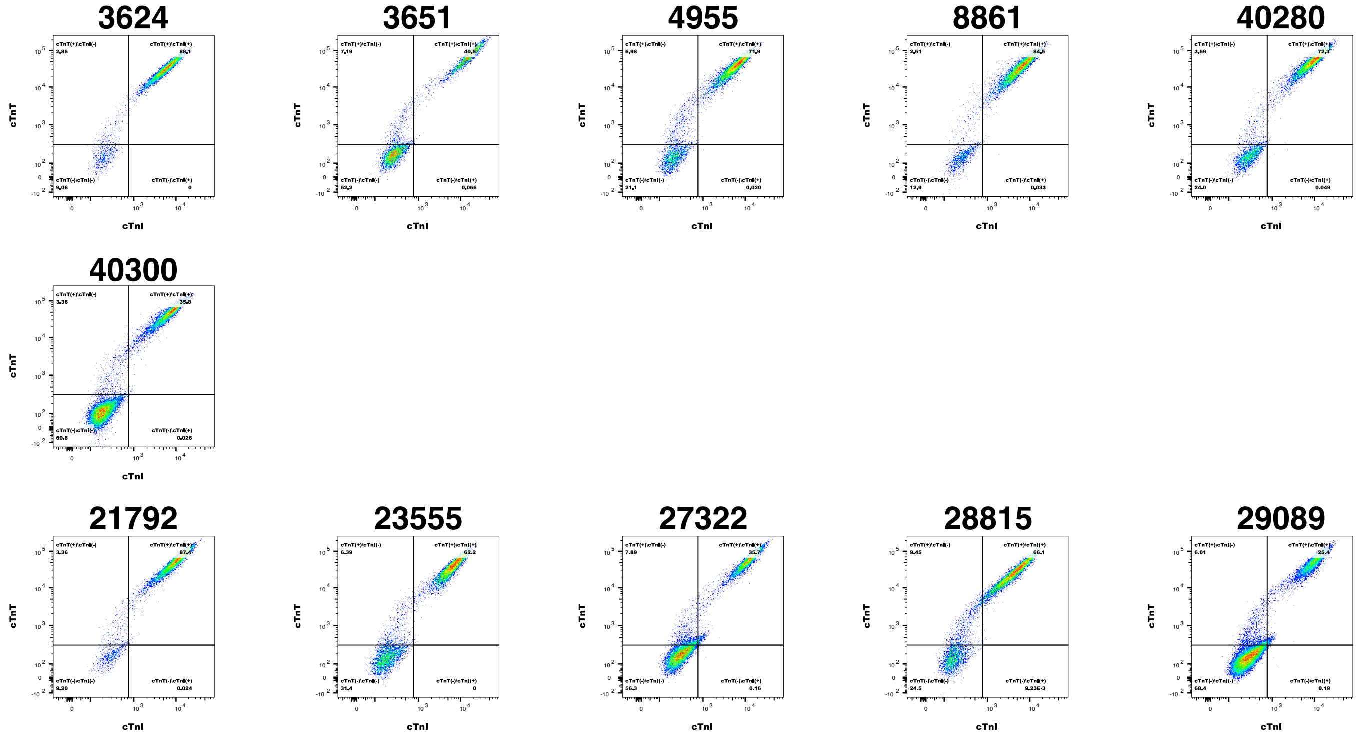

B.

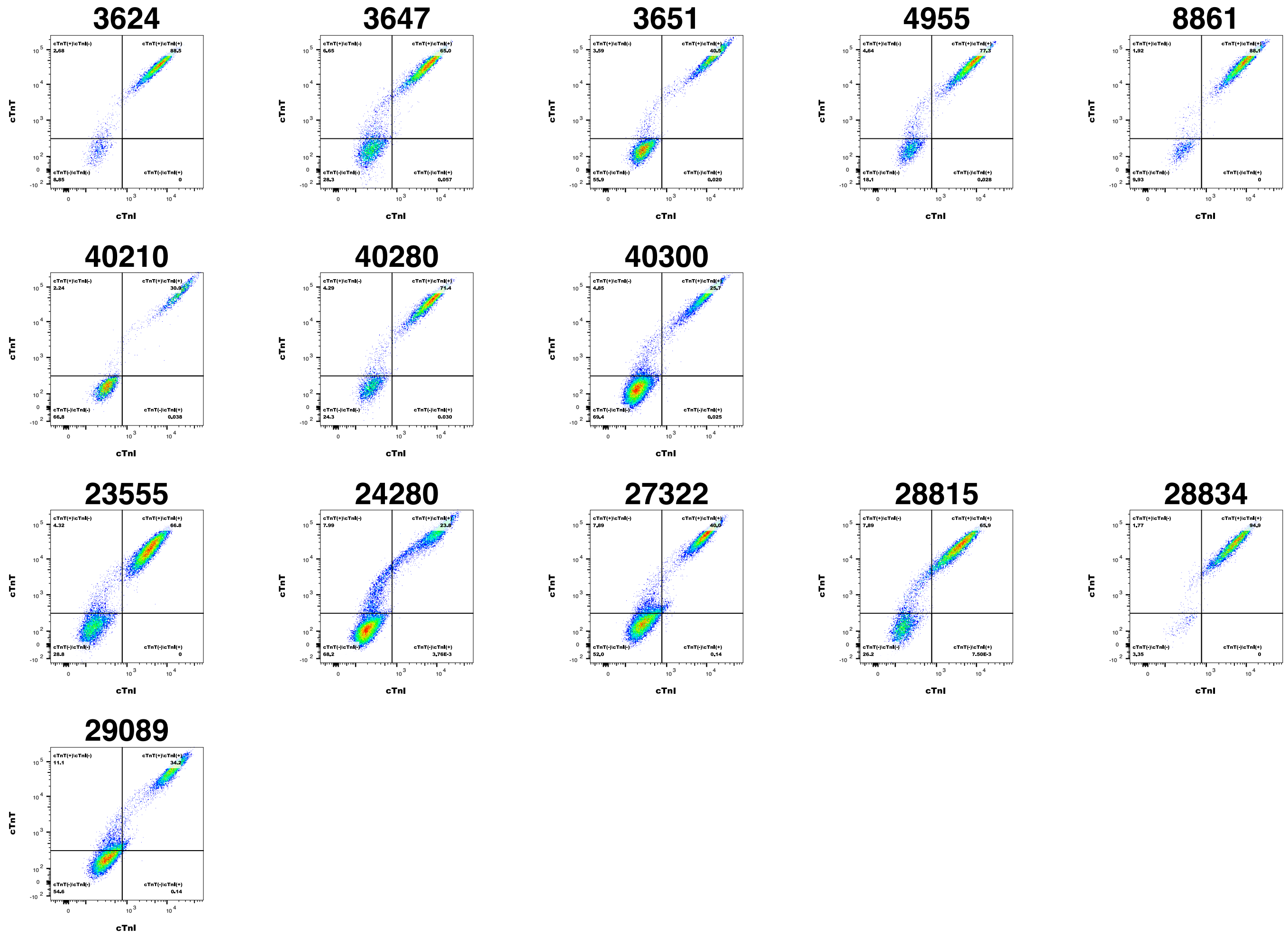

Supplemental Figure 13. Psuedocolor plots showing gating for iPSC-derived cardiomyocytes from batch B at Day 27. Gating and purities are shown for iPSC-derived cardiomyocyte samples collected in batch B at day 27 without T3 treatment (**A.**) and with T3 treatment (**B.**). Plots and purities are shown with dead cells and debris already removed from analysis (methods). iPSC line identifiers designating different individuals are show above each plot, all line and sample info is included in Supplemental Table 1. Fluorescence intensity for cTnT (TNNT2-PE) is shown on the y-axis, fluorescence intensity for cTnI (TNNI3-A647) is shown on the x axis.

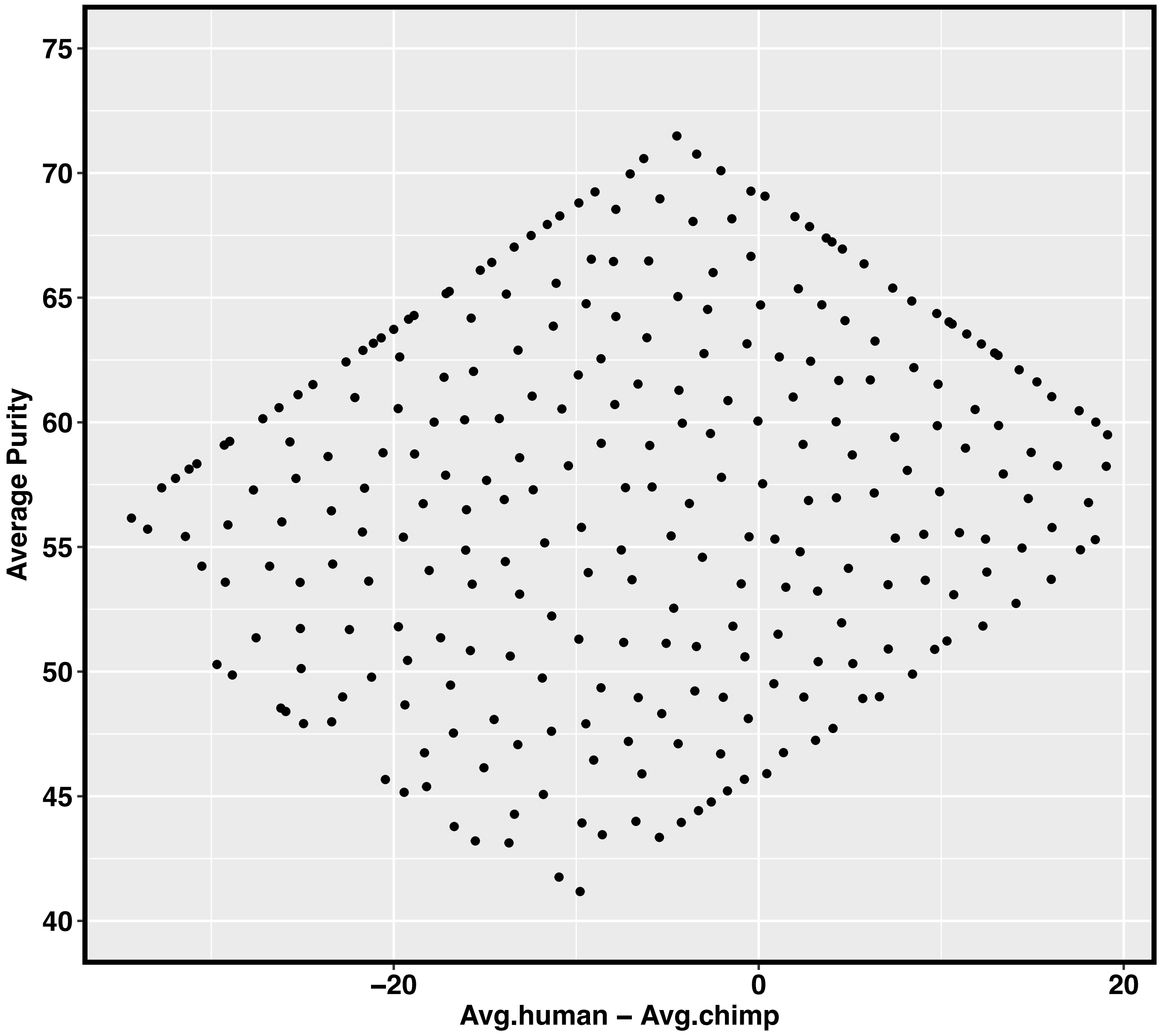

Supplemental Figure 14. Average purity for all samples and difference in purity between species for samples used to estimate the effect of purity on recapitulating interspecies DE genes. A total of 277 different combinations of average purities for 7 chimps and 7 humans were generated to estimate how purity affects recapitulation of interspecies DE patterns identified in heart tissues. The X axis shows the average purity for all samples together, The Y axis shows the difference in average purity between species.
